# The Science of Complex Systems Is Needed to Ameliorate the Impacts of COVID-19 on Mental Health

**DOI:** 10.3389/fpsyt.2020.606035

**Published:** 2020-11-25

**Authors:** Jo-An Atkinson, Yun Ju Christine Song, Kathleen R. Merikangas, Adam Skinner, Ante Prodan, Frank Iorfino, Louise Freebairn, Danya Rose, Nicholas Ho, Jacob Crouse, Vadim Zipunnikov, Ian B. Hickie

**Affiliations:** ^1^Youth Mental Health and Technology, Brain and Mind Centre, Faculty of Medicine and Health, University of Sydney, Camperdown, NSW, Australia; ^2^Computer Simulation and Advanced Research Technologies (CSART), Sydney, NSW, Australia; ^3^Intramural Research Program, National Institute of Mental Health, Bethesda, MD, United States; ^4^School of Computer, Data and Mathematical Sciences, Western Sydney University, Penrith, NSW, Australia; ^5^The Australian Prevention Partnership Centre, Sydney, NSW, Australia; ^6^Department of Biostatistics, Bloomberg School of Public Health, Johns Hopkins University, Baltimore, MD, United States

**Keywords:** systems modeling, simulation, mental health, suicide prevention, COVID-19, recession

## Introduction

### An Exemplar of Scientific Evolution

To assist with proactive and effective responses to the global COVID-19 crisis, the scientific community has been rapidly deploying our most advanced analytic tools to model the dynamics of disease transmission based on existing (albeit imperfect) knowledge, data, and available human and material resources. The multifactorial, multilevel influences on transmission dynamics and the disease's pervasive impact at the individual, community, and global levels have required the use of the analytic techniques of complex systems science, namely, systems modeling and simulation, to forecast the trajectory of the disease under different conditions, to quantify uncertainty, and to inform effective responses (1–3). These methods have been deployed by infectious disease epidemiologists for over a century (4), maturing into a robust interdisciplinary field intersecting mathematics, computational epidemiology, ecology, evolutionary biology, immunology, behavioral science, and public health (5). As a result, there have been numerous advances that have informed policies to control infectious diseases, facilitate epidemic and bioterrorism preparedness, and provide governments with critical tools for managing complexity and weighing alternative responses in the midst of the confusion of an evolving crisis (6–14). The field's commitment to achieving rapid response capability in the face of changing conditions has led to advances in rapid assessment of the impact of the pandemic, and data assimilation methods that combine theory with empirical observations in a continuous knowledge feedback process facilitating continuous hypothesis development, testing, and refinement in the service of more effective decision making (15–19).

### What Can the Mental Health Research Community Learn From This Evolution?

The individual and societal effects of COVID-19 and resulting economic recession are particularly threatening to mental health and well-being, disrupting nearly every aspect of life; familial, educational, vocational, health, and social structures. Most countries, and international agencies, have begun to recognize the unprecedented magnitude of the adverse impact COVID-19 will have on population-based mental health outcomes, mental health services, and suicide risk (20, 21). The global health, economic, and social recovery from this crisis is likely to be prolonged and incomplete, with more disadvantaged communities experiencing particularly severe effects. The mental health impacts will not be limited to the period of the pandemic but will extend with the depth and duration of economic recession. Some governments, particularly in high income countries, are already instituting measures to reduce economic and social hardship, and linked psychological distress, by making large investments in employment and welfare funding, continuity of education and training, housing stability, family support, and improved access to virtual mental health services, resources, and other psychological supports through various new technologies. However, it is unclear whether these responses will be sufficient or sustained for a long enough period to ameliorate the expected tsunami of mental health issues in the post-COVID-19 era.

Unlike in infectious diseases research, systems modeling and simulation has not pervaded mental health research, nor does it underpin advice given to governments. Instead, an epistemological entrenchment primes us to identify and address individual risk factors across the behavioral, social, cultural, economic, environmental, and services spectrum, with little formal (or statistical) recognition of the complex interrelationships between them (22–24). This entrenchment has bound us to a path of linear thinking, and delayed actions, that lacks the agility required to be responsive to a rapidly changing world. Traditional statistical techniques are inadequate for studying complex systems, with feedback loops, threshold effects, and other types of non-linearity bedeviling even more sophisticated analytic techniques such as structural equation modeling and latent class analysis (25). Moreover, traditional approaches focus on looking backwards to generate understanding of the factors that have driven past events, rather than using the current best knowledge and a range of evidence and data sources to look forward and to anticipate and strategically act to mitigate future trajectories. Without adoption of a more advanced approach, the mental health needs of populations—even in times of genuine crisis—may be met with well-meaning acts of government that are reactive rather than strategic, formulated “on the run,” and based on the prioritization of initiatives that have been heavily lobbied or represent “seemingly” good evidence-based investments, but can be ineffective, or even counterproductive (26).

The complex systems modeling techniques long used in infectious disease epidemiology focus on pathways; pathways from states of being susceptible to exposed to infected to recovered (or death), upon which risk factors interact to drive rates of flow between the states based on given probabilities. Accordingly, the mental health research community can similarly articulate complex causal pathways to and from psychological distress, mental ill health, suicidal behavior, and associated functional outcomes. These paths can include complex interactions with service pathways, alongside the dynamics of the broader social context (determinants) of mental health. Such systems models can leverage decades of existing research evidence, local data, and expert knowledge, and could be used to undertake continuous experimentation, assumption testing, and rapid hypotheses refinement through integration with continuous streams of data. This would allow mental health researchers to develop more sophisticated tools for rapid deployment to respond to national and regional threats to mental health (and national mental wealth), by being able to work collaboratively with governments and local planners to answer the critical questions of: “*what combination of responses are required, at what time, in what sequence, targeted at whom, with what intensity, and for how long?*” Embedding systems modeling and simulation in ongoing monitoring and evaluation cycles leverages new data and permits continuous feedback between real-world and modeled mental health service systems to strengthen models over time and secure their role as long-term decision support assets.

Infrastructure to support the collection of sound empirical data will also be important for the development and refinement of models that can accurately forecast longer term mental health and associated educational, vocational, and social outcomes. Alternative assumptions and parameter estimates can produce divergent simulated outcomes. Therefore, reducing the long-term individual and societal costs of the looming mental health epidemic will rely on diverse cross system data to characterize the multi-level influences on mental health and its impacts. The CRISIS initiative is an exemplary effort designed to enable researchers and care providers to examine the extent and impact of life changes induced by the epidemic on the mental health and the behavior of individuals and families across diverse international settings. When combined with geographic, health systems, economic, and other indicators of exposure, such data will enable identification of pre-, peri, and post-COVID-19 demographic, social, and clinical predictors of both short- and long-term impairment and distress induced by the pandemic and its sequelae (http://www.crisissurvey.org/). In addition, new digital technologies now permit the capture of large amounts of clinical, behavioral, social, and cross-sectoral services interaction data from population subgroups most affected to further strengthen the data infrastructure that would be invaluable to efforts to deploy complex systems models to inform mental health policy (27). However, there are important ethical, privacy, and security implications for the capture and use of digital mental health data for research that require consideration and are discussed elsewhere (28).

## Discussion

As a mental health research community we must immediately recognize that we have reached a critical juncture. A continued lack of engagement with systems modeling to advance our understanding of the complex interactions between drivers of mental health and suicide, as well as guide mental health policy, system reform, strategic planning, and operational decision-making will confine us to simplistic conclusions, delayed actions, wrong turns, trial and error, waste and inefficiency, and a lack of agility to be effective in our responses to a rapidly changing world. Without these tools to help us see forward, on what basis do governments make decisions about how to effectively respond? What combination of initiatives or reforms should be prioritized? What targeting, timing, scale, frequency, and intensity of investments are needed? What impacts should we expect from these investments? How long do we need keep programs and initiatives in place? Will there be rebound effects when we remove them? Will there be unintended consequences? The analytic methods of complex systems science provide us with the tools to answer these important questions; without them decision makers will continue to metaphorically fumble around in the dark as they try to make important strategic decisions that will impact people's lives in fundamental ways for many years to come. As a scientific community, we have a responsibility to use the best tools available, to provide decision makers with the insights needed to make proactive and effective investment decisions to mitigate the adverse impacts on mental health of COVID-19 and recession.

As the impending economic and social risk factors rapidly escalate, we cannot wait until the full impact of the crisis is upon us before we mobilize the decision analytic tools needed to inform strategic societal and health system investments that are preventive rather than reactive. As there may be some delay between the emergence of risk factors and the wider population impacts, and the actual impacts will depend on the length and severity of the social and economic disruption and the unique demographic, behavioral, workforce, and service structure and dynamics of a region, now is the time to lay the groundwork (regionally, nationally, and internationally) for these efforts.

**Figure 1:**
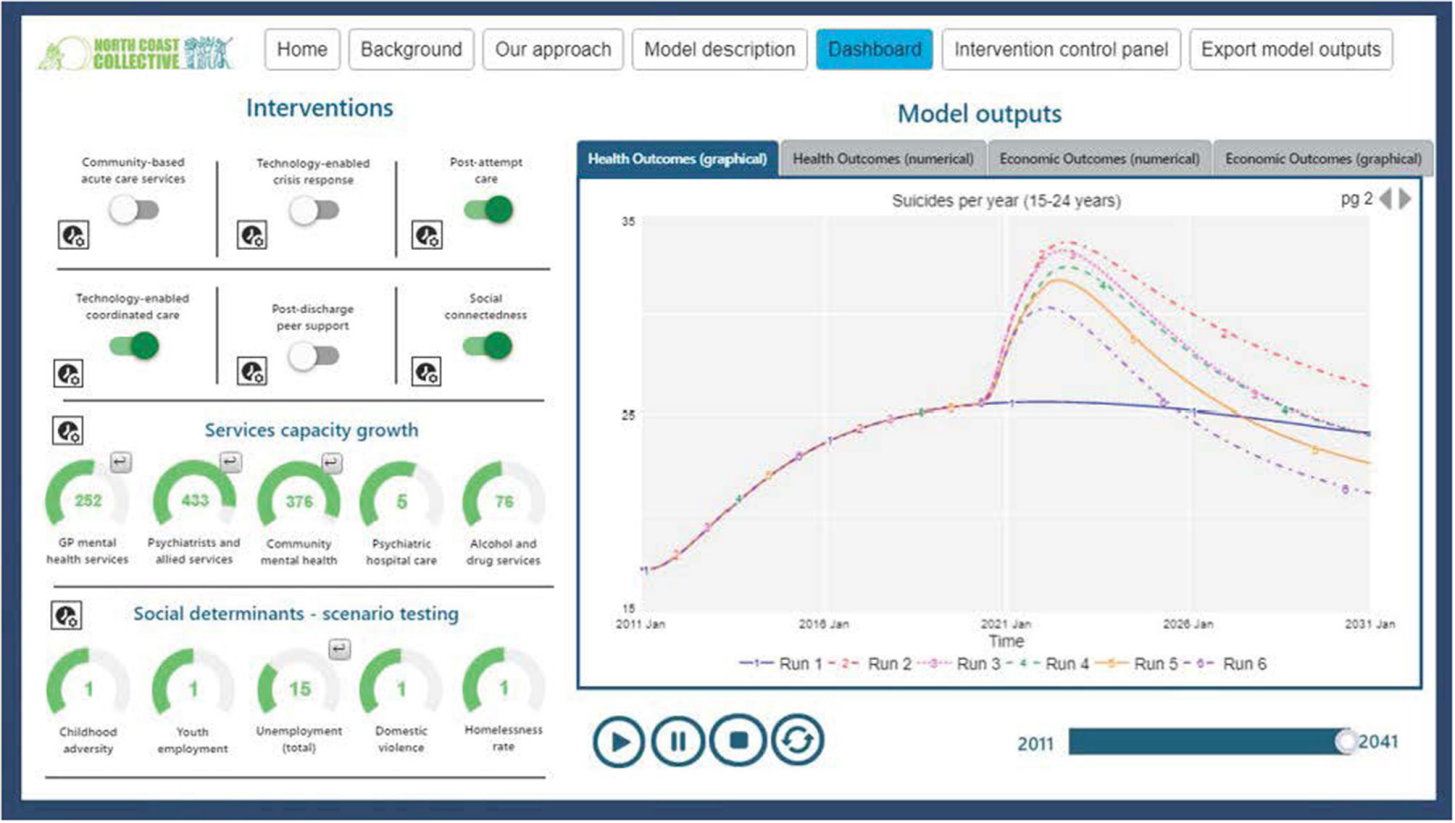
Interactive model interfaces facilitate transparent scenario testing and collective decision making.

Applications of systems modeling in mental health research and practice have already demonstrated value in providing improved decision support capability and a better understanding of the different ways even “evidence-based” interventions can play out in diverse systems and settings. For example, a system dynamics model of suicide prevention developed and validated for a rural and remote population catchment of New South Wales, Australia, demonstrated that some evidence-based interventions were likely to deliver little or no population-level impact [e.g., general practitioner (GP) training], and some combinations were projected to result in an unintended increase in suicidal behavior (e.g., GP training plus mental health education programs) (26). This unintended consequence arising from two “evidence-based” interventions applied in combination is explained by the imbalance they generate in the dynamics of service capacity vs. demand for services which is regionally specific. A range of similar models have been applied to inform mental health services planning, suicide prevention, and to answer long debated questions that are not able to be tested through real world experimentation (23, 29–32). Most recently, early prototype systems models have been developed for the Australian context to inform national and regional policy responses to the mental health consequences of the economic and social effects of the pandemic (33–37). Transparent, and interactive model interfaces (Figure 1) allow regional or national decision makers and stakeholders to run simulations and test alternative scenarios and assumptions, driving more informed debate abouts trade-offs and facilitating consensus building for collaborative action. These processes are essential to the mobilization of appropriate levels of financial, infrastructure and workforce resourcing for rapid system responses to be mounted effectively.

The time to determine the best responses, governance structures, and resources required to address the emerging mental health crisis is now. For these responses to have the best chance at being effective they need to be underpinned by systems modeling work that is transparent, accessible, inclusive, and embedded in continuous monitoring and evaluation frameworks. It is essential to draw on both the deep foundations provided by the field of infectious diseases modeling and the emerging mental health exemplars that have seen modelers and public health decision makers work actively together to formulate sound policy responses. A blueprint for the approach that has already been developed, applied, and deemed to be feasible and valuable, provides a basis for further technical and operational improvements. Good practice guidelines are available to ensure appropriate implementation of systems modeling including considerations of model scope and design, parameterisation, validation, interpretation, and ongoing maintenance (38). Investments in mental health systems modeling and supportive data infrastructure are required immediately to support both the evolution of the field and the realization of a sustainable capability for rapid deployment of advanced decision support tools to inform government investments and actions.

## Author Contributions

J-AA and IH: manuscript drafting. All authors: manuscript concept and critical revision of manuscript for important intellectual content.

## Conflict of Interest

J-AA is both Head of Systems Modelling, Simulation & Data Science at the University of Sydney's Brain and Mind Centre and Managing Director of Computer Simulation & Advanced Research Technologies (CSART). IH reports personal fees from The University of Sydney, Brain and Mind Centre, grants from Australian National Health and Medical Research Council (NHMRC), personal fees from Australian National Mental Health Commission, personal fees from Innowell Pty Ltd, non-financial support from Australian National Mental Health Commission, NMHRS, non-financial support from Australian Department of Health, Million Minds Advisory Panel, non-financial support from Psychosis Australia Trust, from Medibank Clinical Reference Group, outside the submitted work; and The BMC operates early-intervention youth services at Camperdown under contract to headspace: the national youth mental health foundation, which was funded by the Department of Health, Australian Government, and has previously led community-based and pharmaceutical industry-supported (Wyeth, Eli Lily, Servier, Pfizer, AstraZeneca) investigator-initiated research projects or professionally directed education programs focused on the identification and better management of anxiety and depression. The remaining authors declare that the research was conducted in the absence of any commercial or financial relationships that could be construed as a potential conflict of interest.
